# Review of Positive Psychology Applications in Clinical Medical Populations

**DOI:** 10.3390/healthcare4030066

**Published:** 2016-09-07

**Authors:** Ann Macaskill

**Affiliations:** Psychology Research Group, Sheffield Hallam University, Unit 8 Science Park, Sheffield S1 2 WB, UK; a.macaskill@shu.ac.uk; Tel.: +44-0-114-225

**Keywords:** positive psychology interventions, cardiovascular disease, cancer, diabetes, character strengths, health assets, review

## Abstract

This review examines the application of positive psychology concepts in physical health care contexts. Positive psychology aims to promote well-being in the general population. Studies identifying character strengths associated with well-being in healthy populations are numerous. Such strengths have been classified and Positive Psychology Interventions (PPIs) have been created to further develop these strengths in individuals. Positive psychology research is increasingly being undertaken in health care contexts. The review identified that most of this research involves measuring character strengths and their association with health outcomes in patients with a range of different conditions, similar to the position in positive psychology research on non-clinical populations. More recently, PPIs are beginning to be applied to clinical populations with physical health problems and this research, although relatively scarce, is reviewed here for cancer, coronary heart disease, and diabetes. In common with PPIs being evaluated in the general population, high quality studies are scarce. Applying PPIs to patients with serious health conditions presents significant challenges to health psychologists. They must ensure that patients are dealt with appropriately and ethically, given that exaggerated claims for PPIs are made on the internet quite frequently. This is discussed along with the need for more high quality research.

## 1. Introduction

Positive psychology is not a new concept; rather it is grounded in the early work of psychologists such as Jung, Rogers, Alport, and Maslow, who focused on what individuals were capable of achieving and in identifying conditions necessary for psychological growth and well-being. These early pioneers focused more on the clinical application of their theories and empirical support for these theories is relatively sparse. Two clinical psychologists [1] produced a seminal paper proposing that psychologists could now usefully look at how to promote wellbeing in individuals, as this is an aspect that had been largely ignored historically in favor of studying psychopathology. They stressed the necessity for an evidential empirical basis for this work, as it develops, to differentiate it from the earlier work and to ensure that it is theoretically grounded. Positive psychology was to focus on identifying what makes life worth living for individuals. In doing this, the focus would be on three components; positive institutions, positive experiences and emotions, and the positive attributes that individuals possess. Most research has been on the latter, positive attributes possessed by individuals labelled as either, psychological strengths, character strengths or psychological assets. These are the focus of this review.

Psychological strengths can be defined as naturally occurring capacities in individuals that allow them to behave, think or feel in ways that promote their optimal functioning and performance in the pursuit of goals that they value. Researchers [2] have produced a classification of these psychological strengths, the Values-in-Action Classification of Character Strengths (VIA). This identifies 24 character strengths that combine together to produce six higher order virtues, namely, wisdom, courage, humanity, justice, temperance, and transcendence. Most research is at the level of character strengths but it is important to note that the VIA is still being refined to improve its psychometric properties, so the list of strengths may change as evidence of their usefulness accrues [3]. 

Given that positive psychology focuses on assessing well-being, not ill health [4], it is hardly surprising that most research has focused on non-clinical populations. However, this is beginning to change and positive psychology is beginning to be applied in health care contexts, hence the need for this review. Research in positive psychology has been dominated by cross-sectional correlational studies that measure levels of character strengths in individuals and then correlate these with a range of well-being measures and this is also true in clinical populations.

### 1.1 Correlational Studies of Positive Psychology Variables in Medical Populations

Of the papers identified in the literature review, four were recent review papers and, while they did not include Positive Psychology Interventions (PPIs), they provide excellent summaries of current positive psychology correlational studies in cancer and cardiovascular disease. Here, the focus has been almost exclusively on exploring the effect of positive constructs on illness progression and outcomes using correlational studies. The findings of these reviews of correlational studies have been included here to inform researchers, as they can be used to provide a sound empirical basis for researchers to justify the development of PPIs.

The largest number of correlational studies was in the area of cardio-vascular disease. Most of these studies explore the relationship between the positive psychology construct of optimism and various health-related outcomes. This is perhaps unsurprising given that research on optimism predates the current focus on positive psychology, indeed optimism has only recently featured in the Values-in-Action Classification. However, given that research on optimism is well established, it is worth examining in some detail. The most recent systematic review of research on optimism [5] reported on 30 studies involving over 14,000 cardiac patients. Optimism was the main character strength measured in any of the studies and it was included in half the studies reviewed. Hope was included in one study. In terms of well-being, positive affect was the outcome measure in 11 studies. The review concluded that optimism, positive affect and subjective well-being were associated with better health outcomes and reduced mortality even when controlling for depression and negative affect. These findings confirm an earlier review [6] although this only reviewed five studies with mortality as the single outcome variable, but again positive well-being was associated with reduced mortality in cardiac patients. Other earlier reviews of positive psychology constructs in cardiac patients report similar findings [7,8].

For diabetes, the literature review only identified one paper [9] that examined whether positive psychology concepts, in this instance hope and curiosity, were protective factors in relation to the likelihood of a diagnosis of diabetes. This paper also included hypertension, and respiratory tract infection. They measured hope, curiosity, and health behaviours by questionnaire and then examined patient records over a two-year period, either side of the questionnaire administration, to identify diagnosed illness. For all three diseases, higher levels of hope were associated with decreased likelihood of having any of the conditions. Higher levels of curiosity were associated with a lower likelihood of a diagnosis of diabetes or hypertension. Again, these were correlational studies. The conclusion from a review of these correlational studies [10] reported that, while consistent positive associations are found between physical health and positive affect, the quality of the studies is not uniformly high. However, in relation to positive affect and survival from serious illness, the results are inconsistent and the need for further, better quality research is highlighted.

### 1.2 Development of Positive Psychology Interventions

The demonstration of these positive, significant associations between character strengths and well-being in the non-clinical population led to the development of Positive Psychology Interventions (PPIs) to encourage the recognition and utilisation of strengths in individuals with the aim of improving well-being. These PPIs are described as being intended to develop positive cognitions, emotions, and behaviour [11]. Most of these implementations have been with healthy general population samples or students. There is evidence of their effectiveness with these groups, with a review [12] reporting that gratitude visits, gratitude inducing diary exercises and becoming aware of and using strengths in new ways all significantly increased levels of well-being. Another meta-analysis [13] of the effectiveness of PPIs on the general public reported positively but suggested that high quality studies with clinical populations are required as many studies are of poor quality, lacking longer-term follow-up results. A meta-analysis [14] of 51 positive psychology techniques, delivered as additions to group or individual psychotherapy, reported that these additions produced increases in positive affect particularly when delivered to individuals as opposed to groups and where the individuals were strongly motivated to get well. They were also more effective when delivered over longer time periods. Given that this appears to be a developing area, it seems appropriate to conduct a review to identify where positive psychology interventions are being applied for patients with physical illnesses. 

## 2. Method

### 2.1 Literature Search Strategies

Three strategies were followed. Firstly, in December 2015, three major databases were searched for peer-reviewed publications. PsycINFO was used, as it is the major source for psychological studies; Scopus as it is the largest database and includes the PubMed journals from 1996 onwards; and Web of Science as it is good at identifying inter-disciplinary studies, which may be relevant in health care [15]. The aim was to identify studies evaluating positive psychology interventions to promote well-being that involved character strengths, and where the implementation was with clinical samples but not psychiatric samples. The aim was to look at psycho-educational interventions that health psychologists could deliver within their existing skill set. The initial search terms were *positive psychology intervention*, *character strengths*, *clinical population, well-being, life satisfaction, positive emotion*, and *quality of life*. The search was then expanded to include searches with the terms *cancer, cardiovascular disease, diabetes*, and *chronic lung disease*. These conditions were selected, as they are the top four causes of death globally [16]. *Psychological strengths* and *health assets* were also used as alternatives to character strengths in searches. Once suitable papers had been identified, the second strategy involved checking the references in the papers to identify any other relevant studies until no new studies were identified. Thirdly, the author also contributed papers known to her from her reading. 

### 2.2. Selection Criteria

Studies must be quantitative tests of PPIs that include character strengths assessment carried out on adult patients over 18 years, diagnosed with cancer, cardiovascular disease, diabetes or chronic lung disease and be published in peer reviewed journals. Standardized psychometric scales should be used for measuring character strengths and wellbeing should be one of the outcome measures. Psychiatric samples were excluded. *S*tudies had to be in English, accessible, include pre-intervention and post-intervention measures and have a control group, such as treatment as usual, a waiting list or placebo. 

### 2.3. Quality Assessment

The Cochrane Handbook for Systematic Reviews of Interventions [17] was the tool selected to guide the quality assessment of interventions. Seven elements are rated as “high”, “moderate”, or “low”. These are (1) quality control: standardization of the intervention via a manual or published guidance; (2) whether randomization was adequate; (3) comparability of the baseline characteristics of the intervention and control groups; (4) presence of follow-up and completion rates; (5) whether dropout rates are reported and their acceptability; (6) whether there was blind assessment of outcomes or objective outcomes; and (7) whether intention-to-treat analysis was applied. Studies were then evaluated based on a summary score indicating how well that study met each of the criterion, leading to a grading of high, medium, or low quality. For a study to be rated as high quality, all seven criteria had to be met with a follow-up of 90%, moderate when at least four criteria were met and poor when three or less were met. The papers were independently rated by the author and a second experienced researcher. Both agreed the ratings.

## 3. Results

**Figure 1:**
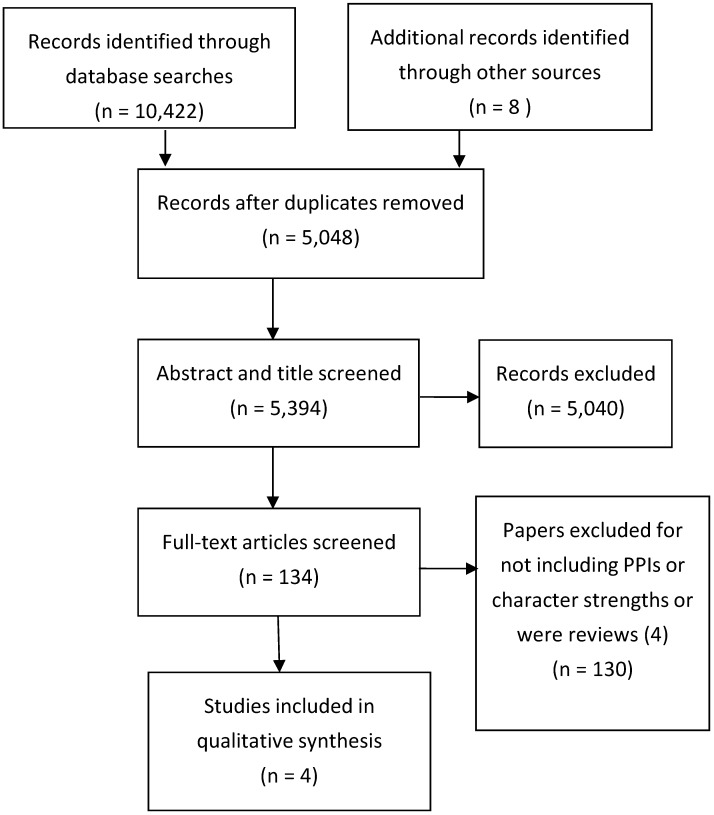
Study selection process [17].

Initially 10,442 papers were identified but, after refining the search terms and removing duplicates, this was reduced to 5048 papers and after screening titles and reading abstracts this was reduced to 134. Further scrutiny of these papers excluded papers that did not include any reference to character strengths related to PPIs and after this only 12 papers were retained. Further application of the criteria reduced this number to eight. It was impossible to apply all the inclusion criteria as standardized psychometric scales were rarely used to measure character strengths and wellbeing was rarely one of the outcome measures. Only one PPI was located for cardiac patients, one for cancer patients, two for adults with diabetes, and none for patients with chronic lung disease. These results are illustrated in the PRISMA [18] diagram in Figure 1.

### 3.1. Cancer

A very recent systematic review identified positive psychology applications in breast cancer [19]. No positive psychology applications were identified for other types of cancer. It was suggested that breast cancer was an appropriate area for PPIs as it is prevalent and disabling, with a risk of relapse, and psychological factors are known to affect the outcome [19]. The recommendation was for positive psychotherapies to be applied as previously described in the literature [14]. Presumably, this advice has been followed as all the PPIs reviewed [19] involved positive psychotherapy. The review concluded that these therapies promote positive changes in the participants but there were issues with the quality of studies and the measures used were often difficult to compare. The systematic review of the literature identified only one PPI for breast cancer that did not involve psychotherapy and this is included in the systematic review [20].

### 3.2. Cardiovascular Disease

While the review identified 30 research studies, utilizing concepts from positive psychology with patients suffering from cardiovascular disease, only one study using a PPI with cardiac patients was identified and it is included in the review [21].

### 3.3. Diabetes

Positive psychology research seems to be rare in patients with diabetes with only three papers located. However, two of these studies involved PPIs for diabetes patients and are therefore included in the review [22,23].

### 3.4. Systematic Review of PPIs

The four papers that included PPIs are described in Table 1. The third paper [22] is included in the table as although only baseline data has been collected; it has been reasonably well designed, improving on the research team’s previous study. It was thought useful to include this as an example of a well-designed study for researchers to examine when developing their own PPIs as only a control group and long-term follow-up is required for a high quality study. Two of the PPIs utilized the same telephone intervention but with patients with cardiovascular disease and diabetes respectively [21,22], stressing the flexibility that telephone interventions provide for patients in terms of reduced travel and increased flexibility of delivery. This mode of delivery is also more economical in terms of patient time and patients get a more personalized intervention. While the contact time for staff delivering the intervention may be slightly longer and thus more costly, the savings made in not having to provide meeting space compensates for this. Staff time is used more effectively as attendance at face-to-face meetings can be patchy, resulting in an inefficient use of staff time.

Flexibility for patients and cost effective delivery was also the rationale for the internet based PPI for diabetes [23]. Patients logged on to the PPI site each week to receive information about what they were to work on the following week. They then received a daily email reminder to complete the exercise and logged on to do this. While systematic comparisons are impossible due to the differing lengths of the interventions and their longevity, it does appear that completion rates for the telephone and internet based interventions were higher than for the face-to-faces sessions. This is something worth examining in future.

The application of the Cochrane Criteria is summarized in Table 2. While in all the interventions aimed to develop positive psychology concepts, the positive psychology concepts themselves were not always assessed. 

For example, one study [21] aimed to develop gratitude, optimism, and kindness but did not measure these variables pre-and post-test despite the availability of psychometrically sound scales for gratitude [24] and optimism [25], although at least it seems that this deficit is being addressed in the proof of concept study [22]. Similarly, one of the PPIs on type 2 diabetes [23] aimed to increase levels of positive affect, gratitude, and mindfulness but did not measure these concepts, although psychometrically sound scales exist for them all respectively [24,26,27]. Although the number of PPIs identified is small, the deficiencies in the three that are complete suggest that they range at best from moderate quality to poor quality. 

## 4. Discussion

The quality of these PPIs was disappointing overall. To find that in the first study [21], in Table 1, that an intervention designed to increase gratitude and positive affect does not include a psychometrically sound measure of either gratitude or positive affect, is difficult to defend. This was also the case in the fourth paper cited [23] with again no psychometrically sound measure of gratitude, positive affect or mindfulness included. There are well validated and extensively used psychometrically sound scales measuring gratitude [24] and optimism [25]. While there is some controversy in the literature about the best scales for measuring different aspects of mindfulness, eight validated scales are available to researchers with guidance on their most appropriate use [28]. On reflection, it may be that researchers who normally focus on measuring physical aspects of health are less familiar with psychological measures and the ready availability of psychometrically sound scales which they can use to directly assess the concepts that interest them.

Similarly, omitting measurement or at least discussion of possible confounding variables such as the life events or treatment effects that may impact on stress levels in patients suffering with breast cancer is a serious deficiency [20]. While two studies [21,23] included randomized control groups, the lack of controls is a serious flaw in the other studies. In health care contexts, there are often serious ethical concerns about using randomized no treatment controls but there is the option to use a treatment as a normal group or a waiting list control. If the intervention is shown to be effective it can be made available to these groups later. The study, which has only published baseline data [22], has included psychometrically sound measures of all the relevant variables, clear selection and adherence to treatment criteria, a pretest-posttest design and a very good description of the intervention for future researchers to follow, but lacks a control group and a long-term follow up to assess whether any changes are maintained. There is clearly a challenge for researchers to develop better PPIs that include all these criteria. 

Finding that positive psychology applications in health are focusing on measuring associations between character strengths and health outcomes is perhaps unsurprising given this is largely the case with positive psychology research on non-clinical populations. The aim of PPIs is ultimately to improve aspects of well-being, by developing strengths and associated positive thinking and reducing stress levels. Applying this to general population samples is relatively unproblematic from an ethical perspective and is perceived as a worthwhile activity. However, when the study population is ill, often with life-threatening conditions, running interventions to make them more grateful, optimistic or happier, seems somewhat insensitive. To make them more applicable to these populations they need to be presented carefully. 

This appears to be beginning to happen within psychotherapy applications as previously discussed [13]. The context of therapy is already supportive and therapists develop a rapport with their clients, making it easier to introduce elements of positive psychotherapy sensitively as ways of increasing the tools that individuals have to support them in their illness. Certainly, patients with chronic recurrent depression reported that character strengths assessment would be a very welcome addition to their treatment, feeling that it provided them with additional resources for coping when they became ill again and it also improved their self-concept to be able to conceptualize themselves as people with strengths [29]. 

Applying PPIs to individuals with serious physical conditions needs to be done very sensitively. Popular applications of positive psychology on a range of websites send the message that if individuals can think positively enough then happiness, personal growth, and well-being is theirs for the taking regardless of circumstances. This can lead to the general public coming to believe that cancer can be overcome by thinking positively, remaining optimistic, being mindful and so on. If an individual is not responding well to their cancer treatment, it is a short step to then thinking it must be because he/she is not trying hard enough to be positive and to suppress negative thoughts. This message is already being disseminated via some popular texts [30]. Others have written about the dangers of exaggerated claims about positive psychology being made, even talking about the tyranny of positive thinking that can lead to individuals who become ill being blamed for their illness [31]. Health psychologists need to guard against exaggerated claims being made for PPIs. Current evidence in health care from a review of mainly correlational studies suggests that character strengths and other health assets such as self-efficacy appear to be associated with better adherence to dietary and exercise health regimes [32]. This is useful knowledge and it suggests that these health behaviors may currently be a more appropriate target for PPIs in health care. 

However, philosophically, there is a mismatch as patients with a physical illness expect health care professionals to take steps to reduce their suffering, not ask them to engage in programs where they are required to work hard at developing their character strengths or thinking more positively. There is a marketing job to be done but before that, there is an ethical requirement to ensure that high quality research is undertaken to ensure that what is being delivered has demonstrated positive benefits. For this, I would argue that more high quality PPIs with non-clinical populations could usefully be run to establish a sounder basis for the effectiveness of existing interventions as reviews of existing studies all comment on a shortage of really high quality RCTS even in the general or student populations [5,7,11,12,13,19]. One particularly critical review of positive psychology as currently applied in cancer care [33] argues for a rethink and an end to exaggerated claims without a sound theoretical and empirical basis. This is a serious challenge for psychologists wishing to incorporate concepts from positive psychology into their health care practice. 

## 5. Conclusions

This review has demonstrated that with clinical medical populations, most applications of positive psychology involve correlational studies with PPIs seldom applied. The one exception is in psychotherapy, where positive psychology techniques are more frequently included in therapy with physically ill patients. There is a shortage of high quality PPIs with both healthy and clinical populations. The design of studies needs to improve, particularly with regard to measuring positive psychology concepts and a sound theoretical base should underpin studies. Researchers need to know where there are positive relationships but also why these relationships are beneficial to health, as well as how they can be fostered. Health psychologists must also actively counteract the exaggerated claims for positive psychology that are increasingly made online and in self-help literature so that patients are appropriately informed. Positive psychology is not a panacea but if carefully developed and delivered, it may provide yet another tool to facilitate well-being for patients with physical health problems. However, there is a real need for well-designed PPIs using standardized psychometric measures and RCT methodology to test whether the benefits found in non-clinical populations can be replicated with a clinical population to similarly improve their well-being.

## Figures and Tables

**Table 1:** Characteristics of included PPIs.

References, First Author, Condition	Study Design	Constructs Targeted, Delivery Mode, Duration	Sample	Sampling method	Outcome Measures	Results	Quality Assessment
[21] Huffman (2011) Cardiovascular Disease	RCT Pretest -posttest design with a control group and treatment as usual group (relaxation)	Gratitude, (3 good things), letter writing, optimism, (best possible self), kindness. First session (45 min) held in hospital, programme explained and first exercise given. Subsequent exercise delivered weekly via a 15-min telephone call to review previous exercise and set new one. Sessions delivered by social worker over 12 weeks and a written manual provided.	Hospitalised cardiac patients *N* = 30 3 equal *n* groups: 1. PPI 2. Relaxation 3. Control undertook an unrelated activity	Self-selecting, randomized to intervention group	Pre-test and post-test scores for happiness *, positive affect *, mental health related quality of life *, anxiety *, depression *.	In PPI group depression, anxiety, happiness, Quality of life showed significantly more improvement from baseline after 8 weeks than in the other two groups.	Medium No direct measure of gratitude, or positive affect. Self-selecting volunteers No longer term follow-up
[20] Rustøen (2011) Breast cancer	Single sample with follow up at 3- and 12- months	Hope, Impact of Events (stress) Hope intervention (Hope-in) Programme 8 weekly sessions lasting 2 h delivered by trained nurses to groups of 5–12 patients in health care setting.	*N* = 195 all diagnosed with breast cancer living at home but requesting professional support via a regional office.	Self-selecting responded to request from health care workers	Pre and post -test scores for hope, Impact of Events scale (stress)	Levels of hope increased Post-intervention and stress levels decreased but these decreases were not maintained at 3- and 12- month follow up.	Medium No control group. No measurement of possible confounding life experience/health variables that may impact on stress levels
[22] Huffman (2015) Types 2 diabetes Intervention delivery designed on Huffman (2011)	Single-arm proof of concept study. Pretest -posttest design.	Optimism, gratitude, positive affect. First session (45 min) held in hospital or on phone, programme explained, and first exercise given. Subsequent exercise delivered weekly via a 15 min. telephone call to review previous exercise and set new one. Sessions delivered over 12 weeks and a written manual provided.	*N* = 15 adults all with diagnosis of diabetes confirmed by a clinician 13 completed	Self-selecting, recruited by researchers at hospital and outpatient diabetic clinics. Had to meet diagnostic criteria and adherence to treatment.	Optimism *, gratitude *, anxiety *, depression *, diabetes distress *, diabetes self-care activities *, health behavior adherence *	Only baseline data reported. Study ongoing. No follow up paper found or response from study team.	Incomplete Good description of intervention but study ongoing.
[23] Cohn, (2014) Type 2 diabetes	Pretest -post-test design with randomised control group from the self-selecting volunteer sample.	Positive affect, gratitude, acts of kindness, mindfulness, incorporated into a package. (Developing affective health to improve adherence to diabetes treatment) Delivered online over 5 weeks. Intervention was a weekly exercise on concept and daily exercises. Participants visited the website daily to record emotions and treatment adherence. Received a daily email reminder. New exercise posted each week. Control group only completed online emotion reporting. Paid for participation.	*N* = 49, all diagnosed with Type 2 diabetes.	Self-selecting, recruited online from a research volunteering site (n = 25) or from a diabetes education centre (n = 28)	Depression, perceived stress, positive & negative affect, diabetes self-efficacy, diabetes distress, diabetes-relevant health behaviours	Post- intervention reductions in depression, No changes in other measures.	Poor as an evaluation of a PPI as no direct measure of gratitude, positive affect, or mindfulness although study aims to develop these strengths. More a feasibility study.

Note * indicates that psychometric scales were used to measure variables.

**Table 2:** Application of Cochrane Criteria to PPIs.

Cochrane Criteria	[20] Huffman (2011) Cardiovascular Disease	[16] Rustøen (2011) Breast Cancer	[22] Huffman (2015) Types 2 Diabetes	[23] Cohn, (2014) Type 2 Diabetes
1.Handbook or written guidance on PPI	1	1	1	1
2. Randomisation adequate	1	1	0	1
3. Comparability of groups	1	0 no control group pre-post design	0 no control group pre-post design	0 no control group pre-post design
4. Longer term follow-up	0	1	0	0
5. Dropout rate given and acceptable	1	0	1 from initial recruitment	1
6. Assessed by objective outcomes	yes, but not all relevant for PPI	1	1 in future	yes but not all relevant ones for PPI
7. Intent-to treat analysis applied	0	0	intended	0
Total scores	4	4	Study continuing and only baseline data available.	3
